# Prospective comparison of CT and ^18^F-FDG PET/MRI in N and M staging of primary breast cancer patients: Initial results

**DOI:** 10.1371/journal.pone.0260804

**Published:** 2021-12-02

**Authors:** Nils Martin Bruckmann, Julian Kirchner, Janna Morawitz, Lale Umutlu, Ken Herrmann, Ann-Kathrin Bittner, Oliver Hoffmann, Svjetlana Mohrmann, Marc Ingenwerth, Benedikt M. Schaarschmidt, Yan Li, Andreas Stang, Gerald Antoch, Lino M. Sawicki, Christian Buchbender

**Affiliations:** 1 Department of Diagnostic and Interventional Radiology, Medical Faculty, University Dusseldorf, Dusseldorf, Germany; 2 Department of Diagnostic and Interventional Radiology and Neuroradiology, University Hospital Essen, University of Duisburg-Essen, Essen, Germany; 3 Department of Nuclear Medicine, University Hospital Essen, University of Duisburg-Essen, Essen, Germany; 4 Department Gynecology and Obstetrics, University Hospital Essen, University of Duisburg-Essen, Essen, Germany; 5 Department of Gynecology, Medical Faculty, University Dusseldorf, Dusseldorf, Germany; 6 Institute of Pathology, West German Cancer Center, University Hospital Essen, University Duisburg-Essen and the German Cancer Consortium (DKTK), Essen, Germany; 7 Institute of Medical Informatics, Biometry and Epidemiology, University Hospital of Essen, Essen, Germany; Medical University of Vienna, AUSTRIA

## Abstract

**Objectives:**

To compare the diagnostic accuracy of contrast-enhanced thoraco-abdominal computed tomography and whole-body ^18^F-FDG PET/MRI in N and M staging in newly diagnosed, histopathological proven breast cancer.

**Material and methods:**

A total of 80 consecutive women with newly diagnosed and histopathologically confirmed breast cancer were enrolled in this prospective study. Following inclusion criteria had to be fulfilled: (1) newly diagnosed, treatment-naive T2-tumor or higher T-stage or (2) newly diagnosed, treatment-naive triple-negative tumor of every size or (3) newly diagnosed, treatment-naive tumor with molecular high risk (T1c, Ki67 >14%, HER2neu over-expression, G3). All patients underwent a thoraco-abdominal ceCT and a whole-body ^18^F-FDG PET/MRI. All datasets were evaluated by two experienced radiologists in hybrid imaging regarding suspect lesion count, localization, categorization and diagnostic confidence. Images were interpreted in random order with a reading gap of at least 4 weeks to avoid recognition bias. Histopathological results as well as follow-up imaging served as reference standard. Differences in staging accuracy were assessed using Mc Nemars chi^2^ test.

**Results:**

CT rated the N stage correctly in 64 of 80 (80%, 95% CI:70.0–87.3) patients with a sensitivity of 61.5% (CI:45.9–75.1), a specificity of 97.6% (CI:87.4–99.6), a PPV of 96% (CI:80.5–99.3), and a NPV of 72.7% (CI:59.8–82.7). Compared to this, ^18^F-FDG PET/MRI determined the N stage correctly in 71 of 80 (88.75%, CI:80.0–94.0) patients with a sensitivity of 82.1% (CI:67.3–91.0), a specificity of 95.1% (CI:83.9–98.7), a PPV of 94.1% (CI:80.9–98.4) and a NPV of 84.8% (CI:71.8–92.4). Differences in sensitivities were statistically significant (difference 20.6%, CI:-0.02–40.9; p = 0.008). Distant metastases were present in 7/80 patients (8.75%). ^18^ F-FDG PET/MRI detected all of the histopathological proven metastases without any false-positive findings, while 3 patients with bone metastases were missed in CT (sensitivity 57.1%, specificity 95.9%). Additionally, CT presented false-positive findings in 3 patients.

**Conclusion:**

^18^F-FDG PET/MRI has a high diagnostic potential and outperforms CT in assessing the N and M stage in patients with primary breast cancer.

## Introduction

Accounting for approximately 12% of new cancer diagnoses every year, breast cancer is considered to be the most frequent cancer in women worldwide and the leading cause of cancer-related death [1]. Treatment concepts and consecutively the survival rate heavily depend on the initial cancer staging. Therefore, accurate imaging-based staging of patients with newly diagnosed breast cancer is playing a pivotal role to determine the optimal treatment management and to minimize potential harmful surgical interventions and extensive systemic therapy [2].

Key points for the initial staging are the detection of tumor manifestations in the contralateral breast, evaluation of locoregional lymph nodes and the detection of distant metastases [3]. Depending on the primary tumor size and the locoregional lymph node status, surgery can extent from a breast-conserving therapy to a complete mastectomy and ipsilateral axillary dissection. In case of validated distant metastases the treatment concept switches to an extensive systemic and most likely palliative therapy [2].

According to the 2018 European Society For Medical Oncology (ESMO) and the 2016 National Comprehensive Cancer Network (NCCN) guidelines a staging, including contrast-enhanced thoraco-abdominal CT and bone scintigraphy [2, 4], is considered in patients with advanced breast cancer (UICC III/IV) and patients with UICC stage II combined with additional risk factors like clinically positive axillary lymph nodes, large tumor size, aggressive biology (HER2neu over-expression, triple negative tumor) or clinical signs/laboratory values suggesting the presence of metastases. Staging in early breast cancer is directed at locoregional disease as patients do not benefit from a whole-body staging, since asymptomatic distant metastases are very rare in early tumor stages [2, 4–6].

Recent studies revealed a superiority of hybrid imaging modalities in detection of distant metastases and in correct malignant lesion rating for breast cancer patients [7–9]. Thus, dual imaging methods have been implemented in international guidelines recommending a ^18^F-FDG PET/CT when conventional methods are inconclusive, in high-risk patients or in patients with newly diagnosed stage III breast cancer [4, 6]. Nevertheless, recent studies demonstrated the superiority of ^18^F-FDG PET/MRI compared to ^18^F-FDG PET/CT in the detection of breast cancer metastases [10]. Especially in combination with a dedicated breast (PET/) MRI may serve as a valuable one-stop-shop alternative for primary staging of breast cancer patients [11–17].

Thus, the purpose of our study was to evaluate the diagnostic potential of CT resembling the current clinical standard compared to ^18^F-FDG PET/MRI for the initial N and M staging of primary breast cancer patients.

## Material and methods

### Patients

This study was approved by the institutional review board of the University Duisburg-Essen (study number 17-7396-BO) and Düsseldorf (study number 6040R) and performed in conformance with the Declaration of Helsinki [18]. All enrolled patients underwent an ^18^F-FDG PET/MRI and contrast-enhanced thoraco-abdominal computed tomography after written informed consent form was obtained. A total of 80 women (mean age: 52.9±11.9, range: 29–79 years) with newly diagnosed breast cancer were enrolled in this prospective study between August 2017 and October 2019. Following inclusion criteria had to be fulfilled: (1) newly diagnosed, treatment-naive T2-tumor or higher T-stage or (2) newly diagnosed, treatment-naive triple-negative tumor of every size or (3) newly diagnosed, treatment-naive tumor with molecular high risk (T1c, Ki67 >14%, HER2neu over-expression, G3). Exclusion criteria were former malignancies in the last 5 years, contraindications to MRI or MRI contrast agents and pregnancy or breast-feeding.

### CT

Thoraco-abdominal multi-slice contrast-enhanced CT were performed in two different CT scanners (Definition Edge and Definition Flash, Siemens Healthineers). The examination was performed in supine position with the arms above the head. Automated tube current modulation and automated tube voltage selection (CareDose 4D and CareKV, Siemens Healthineers) were applied in all examinations. All CT were acquired after intravenous administration of body-weight adapted non-ionic contrast agent with a time delay commonly used in portal venous phase imaging.

### PET/MRI

All ^18^F-FDG PET/MRI examinations were performed on an integrated 3.0-Tesla PET/MRI scanner (Magnetom Biograph mMR, Siemens Healthcare GmbH, Germany) with a mean delay of 67±16 min after ^18^F-FDG injection. Blood samples of all patients were obtained prior to injection of a body-weight adapted dosage of ^18^F-FDG (4 MBq/kg, mean activity 255±45 MBq) to ensure blood glucose levels below 150 mg/dl.

The field of view (FOV) contained the body volume from head to the mid-thigh using a dedicated 16-channel head-and-neck radiofrequency (RF) coil, a 24-channel spine-array RF coil and up to five 6-channel flex body coils. The examination was performed in supine position with head first and arms next to the body. PET acquisition time was 3 minutes per bed position in four or five positions (axial FOV: 25.8 cm, matrix size 344 x 344) and the PET images were performed concurrently with the MRI. The iterative algorithm OSEM (ordered-subset expectation maximization) was utilized for reconstruction of PET images with 3 iterations and 21 subsets and a Gaussian filter with 4-mm full width at half maximum.

For MR-based attenuation correction a coronal 3D-Dixon-VIBE sequence (repetition time (TR) 3.6 ms, echo time 1 (TE1) 1.23 ms, TE2 2.46 ms, slice thickness 3.12 mm, FOV 500 × 328 mm, matrix size 192 ×121) was acquired to create a four-compartment model attenuation map (μ-map), calculated from fat-only and water-only data sets. Subsequently, the following MRI sequences were performed:

1. A transverse T2-weighted (T2w) fat-suppressed half Fourier acquisition single shot turbo spin echo (HASTE) sequence in respiratory medium position and a slice thickness of 7 mm.
2. A transverse diffusion-weighted echo-planar imaging (EPI DWI) sequence (b values 0, 500, 1000) in respiratory medium position with a slice thickness of 5 mm.
3. A transversal T1-weighted (T1w) fat saturated post-contrast Volume-Interpolated Breath-hold Examination (VIBE) sequence after intravenous injection of a gadolinium-based contrast agent (0.2 mmol/kg body weight, Dotarem, Guerbet GmbH, Germany) with a slice thickness of 3 mm.

### Image analysis

All images were interpreted by two experienced radiologists in hybrid imaging in consensus and in random order with a reading gap of at least 4 weeks to avoid recognition bias. CT and ^18^F-FDG PET/MRI images were read in separate sessions. A picture archiving and communication system (Centricity; General Electric Medical Systems, Milwaukee, WI, USA) and a dedicated OsiriX workstation (Pixmeo, SARL, Bernex, Switzerland) were used for image analysis. Lesion count, lesion localization, lesion characterization (benign or malignant) and size as well as the diagnostic confidence of every lesion were assessed (5-point ordinal scale, 1 = very low confidence, 2 = low confidence, 3 = indeterminate confidence, 4 = high confidence, 5 = very high confidence). The malignancy criteria for evaluating the lymph nodes were established based on previous studies, comprising both morphological and metabolic criteria. Following criteria were applied to determine suspicious lymph nodes: a short-axis diameter >10 mm, increased contrast enhancement, spherical configuration, irregular shape, diffusion restriction and focally increased FDG-uptake. At least two of these criteria had to be fulfilled to rate a lymph node as malignant [19, 20]. Criteria for distant metastases were a local invasive growth, central necrosis, contrast enhancement and a typically malignant MR signal like diffusion restriction. In addition, on ^18^F-FDG PET/MRI a visually detectable focal FDG-uptake above background signal was considered as a sign of malignancy. The maximum standardized uptake value (SUVmax) and the maximum diameter were measured in all suspected lesions.

### Reference standard

Due to clinical and ethical standards a histological confirmation of all detected malignant lesions was not applicable and a surrogate reference standard was applied taking into account all follow-up imaging. 148 out of 236 lesions were confirmed histopathologically. 55 lesions were followed-up by CT and 25 lesions by MRI (4±3 months). The remaining 8 lesions were followed-up with sonography and clinical examination, consisting of five benign axillary lymph nodes, two liver cysts and one liver haemangioma.

### Statistical analysis

SPSS Statistics 22 was used for data analysis (IBM Inc., Armonk, NY, USA). All data are presented as mean±standard deviation. Data were analyzed on a per-patient and a per-lesions basis, calculating sensitivity, specificity, positive and negative predictive values. To investigate statistically significant differences between CT and ^18^F-FDG PET/MRI the McNemar chi^2^ test was performed. A Wilcoxon test was used to assess differences between CT and ^18^F-FDG PET/MRI regarding the diagnostic confidence. A p-value of less than 0.05 designated a statistical significance.

## Results

### Patient-based analysis

#### Lymph nodes

**Fig 1:**
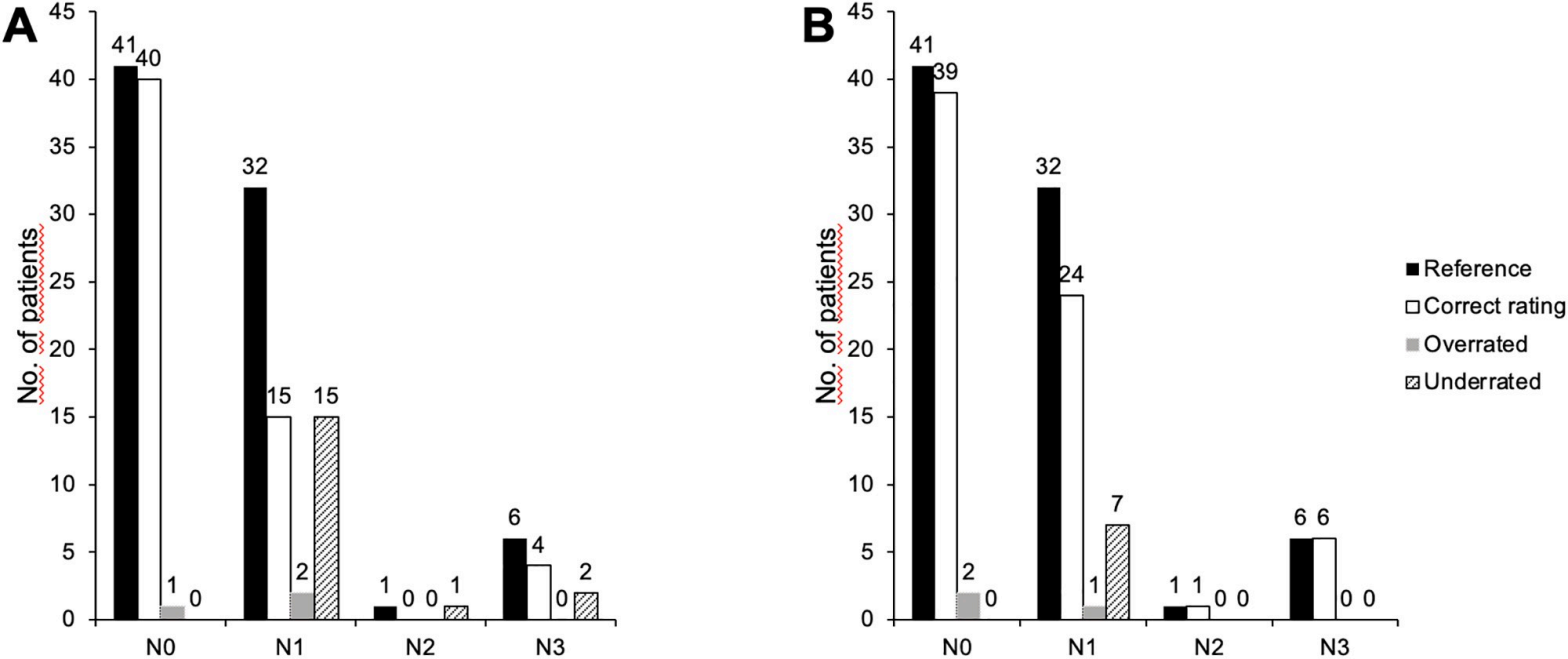
Determination of the lymph node stage in CT (A) and ^18^F-FDG PET/MRI (B).

CT and ^18^F-FDG PET/MRI were concordant for N and M stage in 56 of 80 patients (70%). CT determined the exact N stage correctly in 59/80 (73.75%, 95% CI:63.2–82.1) of the patients (Table 1). The distinction between nodal-positive and nodal-negative patients was rated correctly in 64 of 80 (80%, CI:70.0–87.3) patients by CT. This results in a sensitivity of 61.5% (CI:45.9–75.1), a specificity of 97.6% (CI: 87.4–99.6), a PPV of 96% (CI: 80.5–99.3) and a NPV of 72.7% (CI: 59.8–82.7) (Table 2). In comparison to this the exact N stage was determined correctly by ^18^F-FDG PET/MRI in 70/80 (87.5%, CI: 78.5–93.1) of the patients. PET/MRI yielded a correct classification in nodal-positive and nodal-negative patients in 71/80 (88.75%, CI: 80.0–94.0) of the cases. This results in a sensitivity of 82.1% (CI: 67.3–91.0), a specificity of 95.1% (CI: 83.9–98.7), a PPV of 94.1% (CI: 80.9–98.4) and a NPV of 84.8% (CI: 71.8–92.4). In total, 39 of the patients (48.75%) had a nodal-positive status. 15/39 (38.5%, CI: 24.9–54.1) nodal-positive patients were missed by CT, while ^18^F-FDG PET/MRI missed 7/39 (17.5%, CI: 9.0–32.7) patients. In detail, all missed lymph nodes were stage N1 in both modalities. However, CT underrated two patients with N3 stage according to the reference standard as N1, while PET/MRI was able to detect all of the six patients with N3 stage. Practically, this led to a change of therapy in two patients with expansion of the radiation field after surgery (Fig 1). The McNemars chi^2^ test yielded a significant difference in favor of ^18^F-FDG PET/MRI over CT for determining nodal-positive patients (test statistic = 13.7, p = 0.008) with a corresponding difference in sensitivities of 20.6% (CI: -0.02-40-9). The corresponding difference in specificities did not reach statistical significance (difference 2.4%, CI: -0.06–12.6; test statistic = 0.05, p = 1.0).

**Table 1:** N and M staging on a patient-based analysis.

	CT	PET/MRI	Standard of reference
N stage			
0	40	39	41
1	15	24	32
2	0	1	1
3	4	6	6
Total correct N ratings	59 (73.75%)	70 (87.5%)	80 (100%)
			
M Stage			
0	70	73	73
1	4	7	7
Total correct M ratings	74 (92.5%)	80 (100%)	80 (100%)

Distribution of N and M staging for CT alone and ^18^F-FDG PET/MRI and comparison to the reference standard.

**Table 2:** Diagnostic performance of CT and PET/MRI on a patient-based analysis.

	Locoregional metastases		Distant metastases	
	CT	PET/MRI	CT	PET/MRI
True positive (n)	24	32	3	7
True negative (n)	40	39	71	73
False positive (n)	1	2	3	0
False negative (n)	15	7	3	0
Sensitivity (%)	61.5	82.1	57.1	100
Specificity (%)	97.6	95.1	95.9	100
Positive predictive value (%)	96.0	94.1	57.1	100
Negative predictive value (%)	72.7	84.8	95.9	100
Accuracy (%)	80	88.75	57.1	100

#### Distant metastases

**Fig 2:**
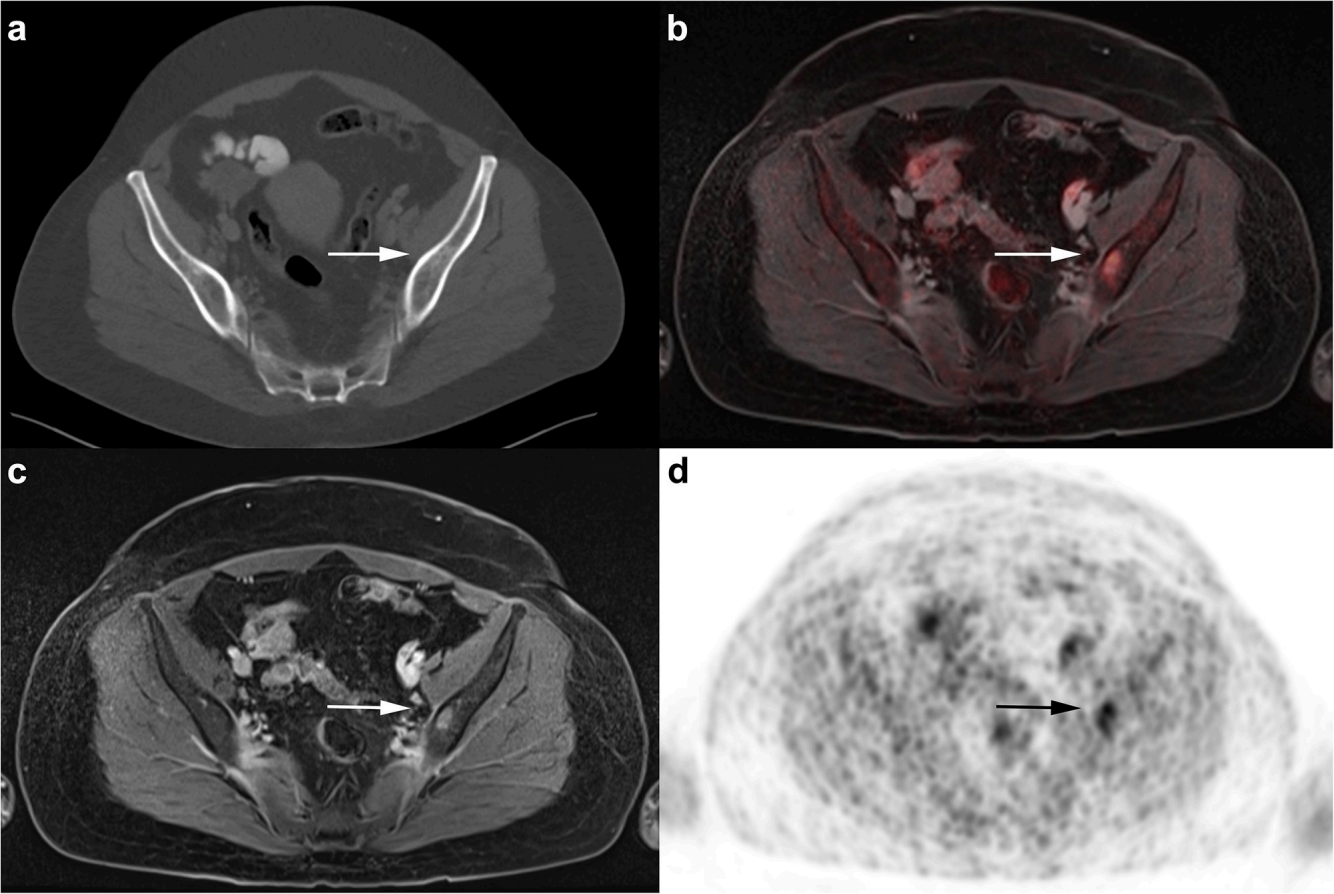
A 45-year old woman with diagnosis of primary breast cancer. No distant metastases were detected in the CT scan (**a**). The subsequently performed ^18^F-FDG PET/MRI shows a bone metastasis in the left iliac bone with contrast enhancement on T1w fs VIBE (**c**) and pathological FDG uptake on PET (**d**) and fused ^18^F-FDG PET/MRI (**b**).

According to the reference standard distant metastases were detected in 7/80 (8.75%) patients. By CT the M stage was defined correctly in 74 of 80 patients (92.5%, CI: 84.6–96.5). In 3 patients, all with bone metastases, CT showed false-negative results (missing 42.9% of the patients with distant metastases). In 2 of these patients the osseous lesions were not visible and in one patient the visible lesions were misinterpreted as simple sclerosis (Fig 2). Furthermore, there were false-positive ratings in 3 patients (4.1%, CI: 1.4–11.4) due to misinterpreted bone degeneration (checked by histopathology), non-specific indurations (checked by CT after 3 month) and several liver hemangiomas (checked by liver-specific MRI). This results in a sensitivity of 57.1% (CI: 25.0–84.2), a specificity of 95.9% (CI: 88.6–98.6), a PPV 57.1% (CI: 25.0–84.2) and a NPV of 95.9% (CI: 88.6–98.6) for the detection of distant metastasis. In comparison to that, ^18^F-FDG PET/MRI was able to detect all malignant lesions without any false-positive findings.

### Lesion-based analysis

A total of 236 lesions were included in the final evaluation, comprising 126 (53.4%) malignant and 110 (46.6%) benign lesions. Table 3 shows the localizations of all malignant lesions, consisting of 90 lymph node metastases and 36 distant metastases. CT detected 192 of 236 lesions (94.5% vs. 81.4%, difference 13.1%, CI: 7.3–18.8), while ^18^F-FDG PET/MRI detected 223 of the 236 lesions.

**Table 3:** Location of all 126 malignant lesions according to the standard of reference.

	Location	Number (n)	Percentage (%)
Distant	Bone metastases	27	21.4
	Lung metastases	4	3.2
	Liver metastases	4	3.2
	Hilar lymph node	1	0.8
			
Locoregional	Lymph node metastases	90	71.4
	Axillary	77	
	Clavicular	7	
	Subpectoral	2	
	Internal mammarian artery	4	
Total		126	100

**Fig 3:**
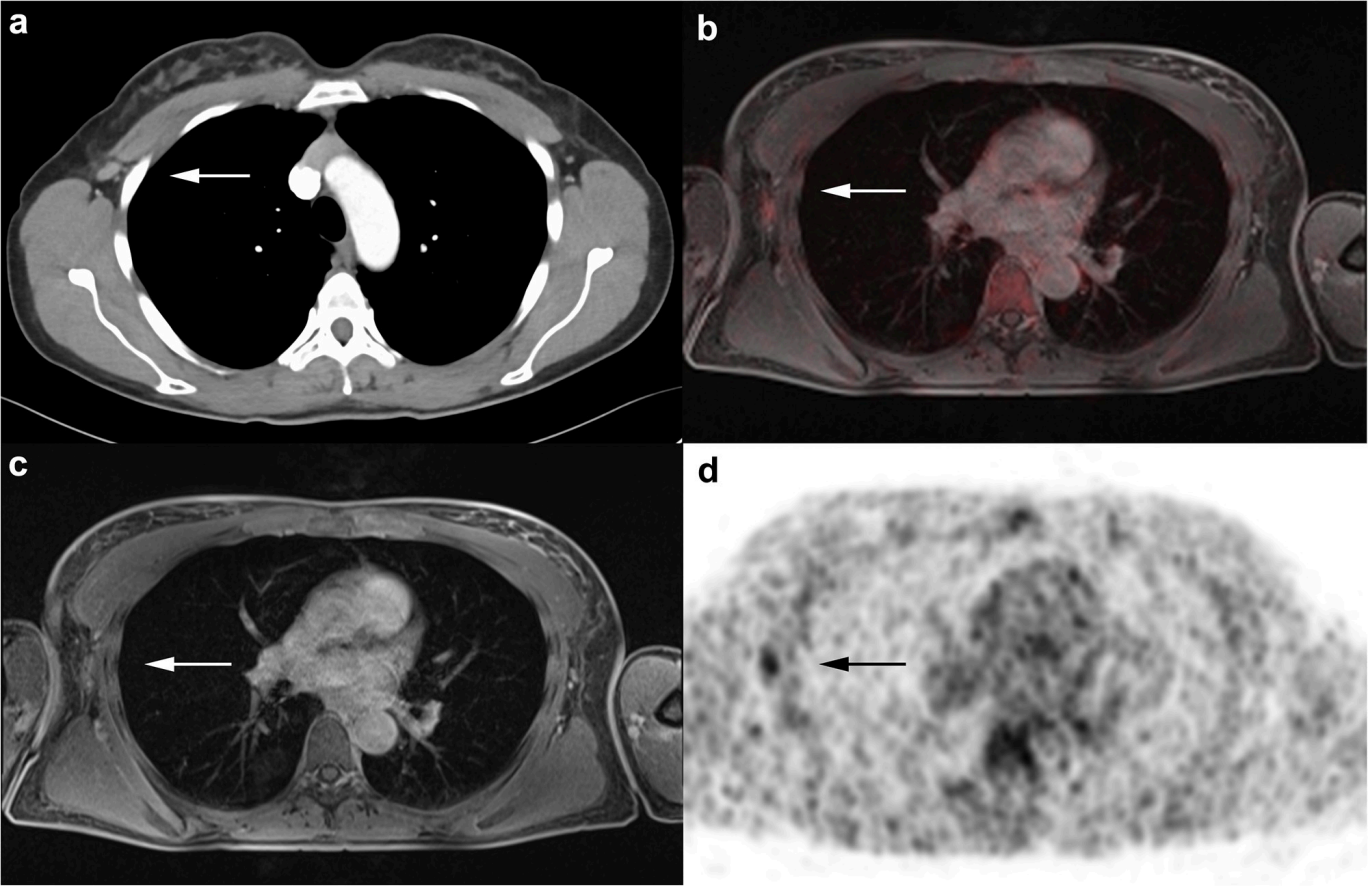
A 48-year old woman with diagnosis of primary breast cancer. Morphologically unsuspicious right axillary lymph node rated as not malignant in the CT scan (**a**). The ^18^F-FDG PET/MRI shows a slight FDG uptake (**b-d**), indicating malignancy. Histopathology proved a tumor infestation.

**Fig 4:**
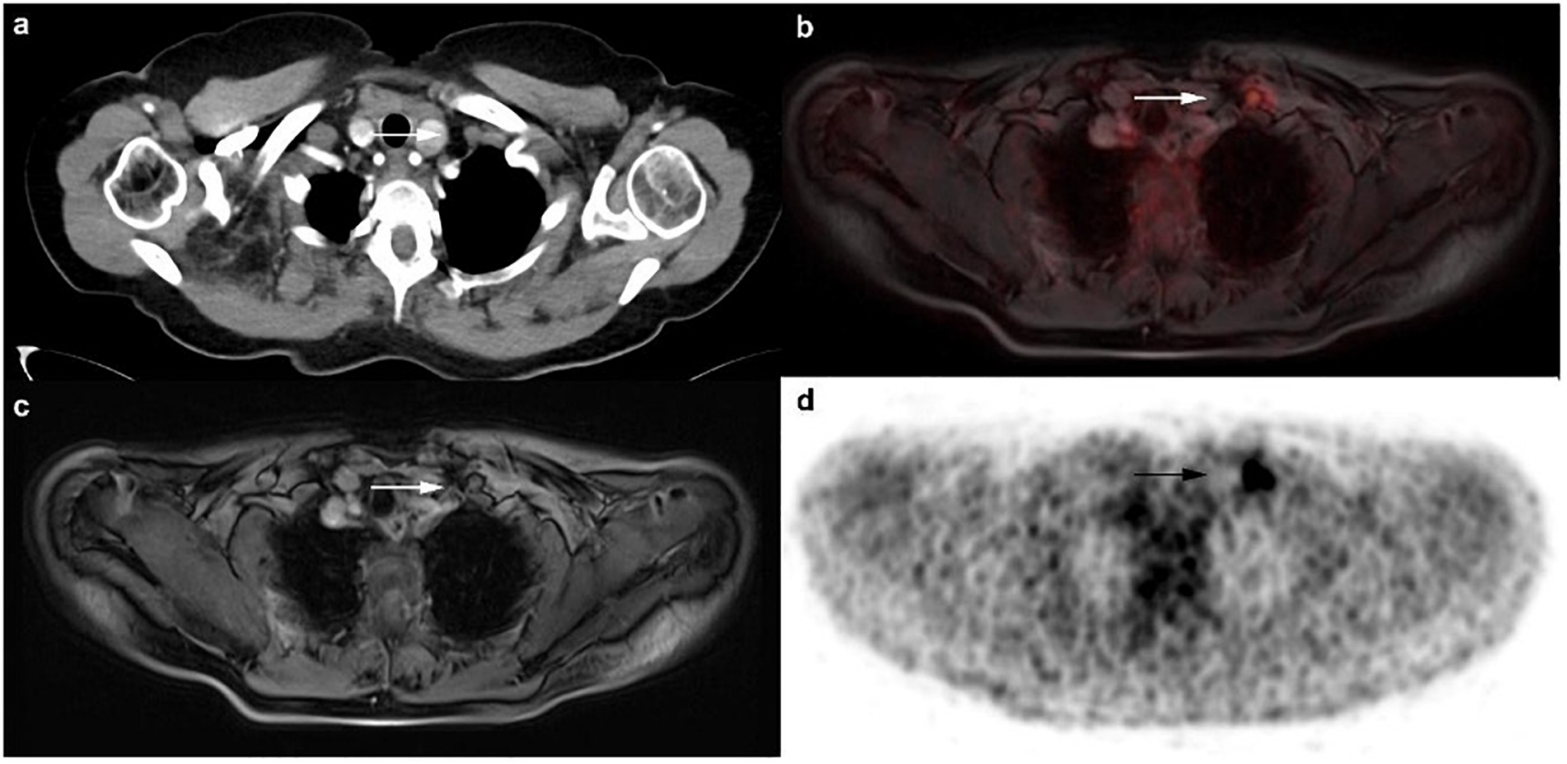
A 72-year old woman with diagnosis of primary breast cancer and lymph node metastases. The reading radiologist did not detect the morphologically inconspicuous left clavicular lymph node in CT scan (**a**). In ^18^F-FDG PET/MRI a clear FDG uptake is visible (**b-d**). Histopathology confirmed malignancy.

CT failed to detect 36 malignant lesions, comprising 19 bone metastases, 2 liver metastases and 1 hilar lymph node metastases as well as 14 locoregional lymph node metastases in axillary (5), mammarian (4), subpectoral (2) and clavicular (3) position (Figs 3 and 4). Furthermore, CT had 27 false-negative ratings, including 23 lymph nodes, which were morphologically unsuspicious and 4 bone metastases, which were evaluated as unspecific sclerosis (Fig 2). There were 12 false-positive findings in CT: 3 bone subsidences, 3 lung indurations, 3 liver haemangiomas as well as a swelling in the contralateral breast and a single axillary and mammarian lymph node were rated as malignant. All these lesions turned out to be benign in histopathology and follow-up examination. All in all CT correctly rated 63/126 of the malignant lesions, resulting in a sensitivity of 50% (CI: 41.4–58.6), a specificity of 88.2% (CI: 80.6–93.1), a PPV of 84% (CI: 74.1–90.6) and a NPV of 76.9% (CI: 68.5–83.6) (Table 4). In contrast to that ^18^F-FDG PET/MRI did not miss any of the 126 malignant lesions. ^18^F-FDG PET/MRI correctly rated 115/126 of the malignant lesions, resulting in a sensitivity of 91.3% (CI: 85.0–95.1), a specificity of 97.9% (CI: 92.8–99.4), a PPV of 98.3% (CI: 94.0–99.5), and a NPV of 89.6% (CI: 82.4–94.1) (Table 4). False-negative ratings were due to axillary lymph nodes with small lesion size and weak FDG-uptake. There were two false-positive findings in ^18^F-FDG PET/MRI, because two axillary lymph nodes demonstrated an increased suspicious FDG-uptake. There was a significant difference for correct rating of lesions as malignant between CT and ^18^F-FDG PET/MRI (63 vs. 115 out of 126 lesions, 50% vs. 91.3%, difference 41.3%, CI: 30.7–50.6; test statistic = 12.1, p<0.0001). The exact division between locoregional lymph node metastases and distant metastases can be seen in Table 4.

**Table 4:** Diagnostic performance of CT and PET/MRI on a lesion-based analysis.

	All lesions		Locoregional metastases		Distant metastases	
	126/236		90/147		36/89	
	CT	PET/MRI	CT	PET/MRI	CT	PET/MRI
True positive (n)	63	115	53	79	10	36
True negative (n)	90	95	49	54	41	41
False positive (n)	12	2	2	2	10	0
False negative (n)	27	11	23	11	4	0
Missed benign (n)	8	13	6	1	2	12
Missed malignant (n)	36	0	14	0	22	0
Sensitivity (%)	50	91.3	69.7	87.8	27.8	100
Specificity (%)	88.2	97.9	96.1	96.4	80.4	100
PPV (%)	84	98.3	96.4	97.5	50	100
NPV (%)	76.9	89.6	68.1	83.1	91.1	100
Accuracy (%)	64.8	89	69.4	90.5	57.3	86.5

### Diagnostic confidence

The diagnostic confidence for distinguishing between benign and malignant lesions was significantly higher in ^18^F-FDG PET/MRI than in CT (3.9±0.7 vs. 3.2±0.7, p<0.0001). Comparing the diagnostic confidence regarding malignant lesions only, containing distant and locoregional metastatic lesions, differences between the two modalities were even higher (4.1±0.7 vs. 3.3±0.7, p<0.0001).

## Discussion

The present study shows the superiority of ^18^F-FDG PET/MRI for the primary N and M staging of breast cancer patients compared to ce-CT. Due to an increasing attention to reach accurate staging prior to therapy, the diagnostic algorithm of breast cancer has changed in recent times. The basic staging with chest x-ray and abdominal sonography has been replaced by thoraco-abdominal CT and bone scintigraphy [2, 4]. Therefore, the current international guidelines recommend a generalized thoraco-abdominal staging with CT and scintigraphy in patients with advanced breast cancer (UICC III/IV) and patients with additional risk factors, since the likelihood of distant metastases is increased with positive lymph node findings and an aggressive tumor biology [5, 21, 22]. This amendment to international guidelines [5, 21] is not based on studies designed specifically for this purpose, but was determined with an expert consensus based on the sensitivities and specificities for lesion detection of each modality. The aim is to improve the detection of distant metastases, since the presence of metastases leads to a completely different therapy regime.

CT is the simplest and most cost-effective alternative for a thoraco-abdominal staging, but in accordance with the current state of science, the sentinel lymph node biopsy and histological work-up is still the clinical standard of choice for determining a lymph node involvement and prove distant metastases. Patients confirmed as nodal-positive are surgically treated undergoing an axillary lymph node dissection (ALND) in a further intervention. A preselection of which patients receive a sentinel lymph node biopsy is currently carried out by clinical examination, sonography, conventional mammography and breast MRI. However, these methods are far from serving as a real alternative to biopsy. So the sentinel lymph node biopsy is very generously performed in patients with the slightest suspicion of a lymph node involvement [23–26].

Hybrid imaging modalities have proven to be advantageous in cancer staging in comparison to conventional imaging techniques [27–29], but these also cannot compete with the biopsy according to the current state of research [26]. However, a better preselection of nodal-positive patients may result in fewer surgeries, avoiding prior lymph node biopsy before ALND. Nevertheless, a general recommendation for the use of hybrid imaging modalities is not given, based on the 2015 and the 2018 European Society For Medical Oncology (ESMO) and the 2018 National Comprehensive Cancer Network (NCCN) guidelines. However, a systemic staging with ^18^F-FDG PET/CT is considered for patients with inconclusive results in conventional imaging and in high risk patients [4–6].

With regard to the correct determination of the N stage, there were huge differences between CT and ^18^F-FDG PET/MRI visible in this study in favor of the ^18^F-FDG PET/MRI. ^18^F-FDG PET/MRI showed a significantly higher sensitivity on a patient-based and a lesion-based analysis and thus demonstrated a higher accuracy of detecting the N stage. In addition, especially the undervaluation of lymph node stage in CT had a direct impact on treatment in two patients, since radiation field had to be expanded after ^18^F-FDG PET/MRI was done.

Although, CT represents the standard in primary breast cancer staging according to the guidelines, this is the first study comparing the diagnostic potential with ^18^F-FDG PET/MRI. However, many studies have already compared ^18^F-FDG PET/CT with conventional imaging techniques. In terms of locoregional lymph node involvement, many authors describe a clear added benefit of ^18^F-FDG PET/CT. Bitencourt et al. emphasize a significant superiority of ^18^F-FDG PET/CT in comparison to conventional imaging especially in providing information on extra-axillary and not enlarged lymph nodes [27, 30–32]. These statements are in line with the results of our study, in which the CT failed to detect 4 not enlarged axillary lymph node metastases and 12 lymph node metastases in mammarian, subpectoral and subclavian position (Fig 4). Mahner et al. [33] also described a clear advantage of hybrid imaging, indicating the sensitivity of CT for the determination of axillary, supraclavicular and mediastinal lymph nodes with 53%, 40% and 31%, compared to PET with 86%, 84% and 96%. In accordance with our results, these studies show that the number of biopsies can be reduced in the future by considerably improving the sensitivity in the detection of lymph node metastases, even if biopsy still remains the gold standard. The more accurate assessment of N stage can ultimately also directly influence therapy, for example in the adaption of the radiation field.

In the assessment of the M stage, our study determined a clear superiority of ^18^F-FDG PET/MRI in comparison to CT. While the CT missed metastasis in 3 patients and also provided false-positive findings in 3 other patients, the ^18^F-FDG PET/MRI was able to detect all of the seven patients with distant metastases without any false-positive findings. These results are consistent with the results from earlier studies. For example, Hildebrandt et al. compared the diagnostic value of ^18^F-FDG PET/CT, contrast-enhanced CT and bone scintigraphy in 100 women with suspected recurrence of breast cancer. The study suggests that PET/CT has a greater accuracy than conventional imaging techniques in this patient group [29, 34]. These results are also supported by a study of Gajjala et al., rating PET/CT as more accurate than conventional imaging techniques for staging locally advanced breast cancer in a cohort of 61 patients. A direct comparison of ^18^F-FDG PET/MRI and CT in recurrent breast cancer is also provided by the study of Sawicki et al. [7]. In this study with 21 patients with suspected breast cancer recurrence ^18^F-FDG PET/MRI offered the highest diagnostic performance and outperformed both CT and PET/CT. Especially the CT alone scored poorly in this study, missing about 30% of malignant lesions (PET/CT: 3.4%; PET/MRI: 0%). On the other hand, there are also studies that have not explored any advantage of hybrid imaging in primary breast cancer patients. In the study of Monzawa et al. with 50 patients suffering from invasive breast cancer no superiority in diagnostic performance of ^18^F-FDG PET/CT in comparison to ultrasonography and contrast- enhanced CT could be determined [35]. Furthermore, it has been confirmed by a whole series of studies that ^18^F-FDG PET/MRI is superior to ^18^F-FDG PET/CT in the detection of breast cancer metastases [20, 36, 37]. Only in the overall detection and characterization of lung lesions ^18^F-FDG PET/MRI gives worse results so far, caused by the limited ability of MRI to detect small lung lesions [38, 39]. However, a superiority of CT over ^18^F-FDG PET/MRI in terms of detection of pulmonary lesions could not be determined in our study. Summarizing, with regard to distant metastases, the performance of PET/MRI can thus have a fundamental influence on the therapy regime for some patients and has to be considered as a helpful diagnostic tool, due to the high sensitivity in distant lesion detection. Taking into account the results of former studies, however, a histological confirmation is still necessary to ensure tumor infestation.

In addition to the direct detection of suspicious lesions, the diagnostic or interpretation confidence of the modalities, with which a lesion can be classified as benign or malignant, is of great interest. This study confirms, that ^18^F-FDG PET/MRI has a great advantage in comparison to CT in the definitive assessment of a suspicious lesion, facilitating the final diagnosis for the reading radiologist. This advantage is primarily due to the glucose uptake of tumorous lesions, which can thus be assessed as malignant and therefore reduce the uncertainty of the radiologist in comparison to conventional imaging techniques [40].

An additional benefit of using PET/MRI is a potential reduction of ionizing radiation, when compared to CT or even PET/CT. This is particularly relevant in the primary staging of breast cancer, since the tumor tends to occur more often in younger patients compared to other cancer entities [41, 42]. In addition, with PET/MRI an imaging of the head is directly acquired. In our study, however, this did not result in any advantage since no relevant findings were discovered.

This study has some limitations. In conformity with previous studies, a modified reference standard had to be applied, based on follow-up imaging for lesions without a histological sampling, since management of advanced tumor stages does not necessarily require a histological sampling of all detected malignant lesions [7, 11, 43].

### Conclusion

In conclusion, the present study shows that ^18^F-FDG PET/MRI has a high diagnostic potential and outperforms CT in assessing the N and M stage in patients with primary breast cancer. Despite the advantages of CT such as availability, costs or acquisition speed, this study together with present data should provide cause of discussion, regarding the current recommendations for primary staging in breast cancer guidelines.

## Abbreviations

AC: Attenuation correction
ALND: Axillary lymph node dissection
CI: Confidence interval
CT: Computed tomography
DWI: Diffusion-weighted imaging
EPI: Echo-planar imaging
ESMO: European Society For Medical Oncology
FDG: Fluorodeoxyglucose
FOV: Field of view
FWHM: Full-width at half maximum
HASTE: Half Fourier acquisition single shot turbo spin echo
HER2: Human epidermal growth factor receptor 2
MRI: Magnetic resonance imaging
NCCN: National Comprehensive Cancer Network
OSEM: Ordered-subset expectation maximization
PET: Positron emission tomography
RF: Radiofrequency
VIBE: Volume interpolated breath-hold examination
WHO: World Health Organization
